# Sterile alpha motif and histidine/aspartic acid domain-containing protein 1 (SAMHD1)-facilitated HIV restriction in astrocytes is regulated by miRNA-181a

**DOI:** 10.1186/s12974-015-0285-9

**Published:** 2015-04-08

**Authors:** Sudheesh Pilakka-Kanthikeel, Andrea Raymond, Venkata Subba Rao Atluri, Vidya Sagar, Shailendra K Saxena, Patricia Diaz, Semithe Chevelon, Michael Concepcion, Madhavan Nair

**Affiliations:** Department of Immunology, Herbert Wertheim College of Medicine, Florida International University, AHC-1, 418A, 11200 SW 8th Street, Miami, FL 33199 USA; Institute of NeuroImmune Pharmacology, Herbert Wertheim College of Medicine, Florida International University, AHC-1, 418A, 11200 SW 8th Street, Miami, FL 33199 USA; Center for Personalized Nanomedicine, Herbert Wertheim College of Medicine, Florida International University, AHC-1, 418A, 11200 SW 8th Street, Miami, FL 33199 USA; CSIR-Centre for Cellular & Molecular Biology, Uppal Road, Hyderabad, 500007 India

**Keywords:** SAMHD1, HIV, Astrocytes, miRNA

## Abstract

**Background:**

Although highly active antiretroviral therapy (HAART) has significantly reduced the morbidity and mortality in HIV patients, virus continues to reside in the central nervous system (CNS) reservoir. Hence, a complete eradication of virus remains a challenge. HIV productively infects microglia/macrophages, but astrocytes are generally restricted to HIV infection. The relative importance of the possible replication blocks in astrocytes, however, is yet to be delineated. A recently identified restriction factor, sterile alpha motif and histidine/aspartic acid domain-containing protein 1 (SAMHD1), restricts HIV infection in resting CD4^+^T cells and in monocyte-derived dendritic cells. However, SAMHD1 expression and HIV-1 restriction activity regulation in the CNS cells are unknown. Though, certain miRNAs have been implicated in HIV restriction in resting CD4^+^T cells, their role in the CNS HIV restriction and their mode of action are not established. We hypothesized that varying SAMHD1 expression would lead to restricted HIV infection and host miRNAs would regulate SAMHD1 expression in astrocytes.

**Results:**

We found increased SAMHD1 expression and decreased miRNA expression (miR-181a and miR-155) in the astrocytes compared to microglia. We report for the first time that miR-155 and miR-181a regulated the SAMHD1 expression. Overexpression of these cellular miRNAs increased viral replication in the astrocytes, through SAMHD1 modulation. Reactivation of HIV replication was accompanied by decrease in SAMHD1 expression.

**Conclusions:**

Here, we provide a proof of concept that increased SAMHD1 in human astrocytes is in part responsible for the HIV restriction, silencing of which relieves this restriction. At this time, this concept is of theoretical nature. Further experiments are needed to confirm if HIV replication can be reactivated in the CNS reservoir.

## Introduction

HIV-1 infection of astrocytes results in an initial productive, non-cytopathogenic infection that diminishes to a poorly productive, persistent infection [1,2]. Even though astrocyte infection is ‘restricted,’ their infection results in cellular dysfunction, contributing to the development of HIV-associated neurocognitive disorders (HAND) [3]. Though astrocytes lack surface CD4 receptor, astrocytes get infected by a CD4-independent viral entry, through the mannose receptor that is expressed on the surface of astrocytes [4-6]. Infected astrocytes initiate productive infection of HIV-permissive blood-derived cells in co-culture experiments [7,8]. Although potent highly active antiretroviral therapy (HAART) suppresses plasma viremia to undetectable levels [9], it is unable to eliminate the virus from quiescent reservoirs [10-12] and/or from sanctuaries like the brain [13-15]. The astrocytic reservoir protected from HAART is capable of initiating new infections upon treatment discontinuation and constitutes an important hindrance to HIV clearance from the central nervous system (CNS) [7,16,17].

Deciphering the innate molecular mechanisms that restrict HIV replication and establish persistent reservoirs is essential for developing strategies to reactivate the reservoir. Overcoming the blocks in HIV replication can lead to the upregulation of the gene expression, making these cells available to immune surveillance and HAART targeting. Efficient replication is blocked in astrocytes at different steps of the HIV life cycle [1,8,18]. Although several pre-transcriptional and post-transcriptional mechanisms are shown to contribute to HIV latency, whether SAMHD1 contributes to the viral restriction in astrocytes is not known [19,20].

Specific ‘intrinsic host restriction’ factors restrict virus replication in mammalian cells. Some cells constitutively express these factors, and in others, they are induced by interferons as part of the innate immune response. Apolipoprotein B mRNA-editing enzyme 3G (APOBEC-3G) [21], bone marrow stromal cell antigen 2 (BST-2) [22], tripartite motif protein 5 alpha (TRIM-5α) [23], and cellular miRNAs [24] are examples of restriction factors targeting HIV-1. SAMHD1, a recently identified nuclear protein restriction factor, hydrolyzes cellular deoxynucleotide (dNTP) pools and hampers retrovirus reverse transcription [25]. Due to the limited deoxynucleotide substrate availability, SAMHD1 inhibits HIV infection of myeloid cells and naïve CD4^+^T cells [26,27]. However, SAMHD1-mediated restriction has not been explored in CNS cells. We hypothesized that increased expression of SAMHD1 restricts HIV infection in astrocytes. In this study, we compared the expression of SAMHD1 in astrocytes and microglia and correlated with the HIV replication.

Some studies have shown that SAMHD1 phosphorylation status correlates with the restriction activity but not its dNTPase activity [28]. However, little is known about the post-transcriptional regulation of SAMHD1 expression. MicroRNAs have been implicated in the HIV gene expression and latency in CD4^+^T cells [29-31]. It is not known whether miRNA modulation plays a role in HIV-1 restriction in astrocytes, and whether miRNA regulates SAMHD1 gene expression. Hence, in this report, we also examined SAMHD1 regulation by miRNA. Here, we report that astrocytes display higher SAMHD1 expression compared to microglia, which is in part accountable for HIV restriction in the astrocytes and miR-181a regulates SAMHD1.

## Results

### HIV infection in astrocytes is restricted

**Figure 1:**
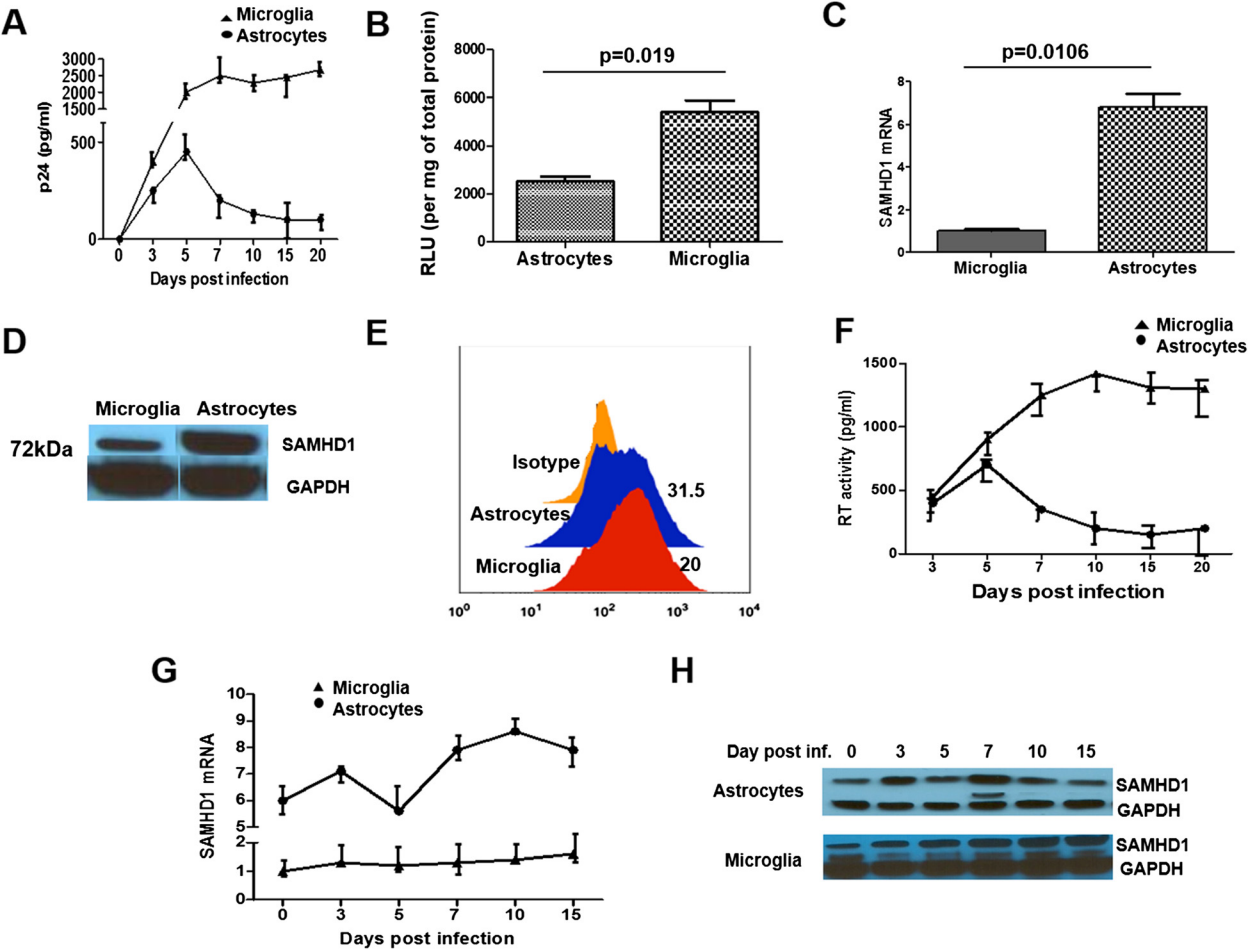
**Restricted HIV replication kinetics correlates with SAMHD1 expression. (A)** 1 × 10^6^ cells/ml astrocytes and microglia were infected with HIV-1_BAL_ (100 ng p24) for overnight, washed and replenished with fresh medium. Virus replication kinetics is quantitated in cell culture supernatant over a 20-day time course, by the production of p24 antigen. **(B)** Cells were infected with the HIV-luciferase pseudovirus (20 ng) for 3 days, harvested, washed with PBS and lysed. Luciferase concentration in whole cell lysates was determined. **(C)** RT-PCR based quantification of SAMHD1 mRNA levels. Total cellular RNA was extracted from microglia and astrocytes (infected with HIV-1_BAL_) and amplified by specific primer for SAMHD1 gene expression. All data was normalized to GAPDH. Data shown represents fold change in mRNA levels. **(D)** SAMHD1 protein expression was detected by immunoblotting with SAMHD1-specific antibodies in corresponding cell lysates. Equal amounts of total protein (30 μg) of the lysates from both cells were loaded. GAPDH was used as a loading control. **(E)** Intracellular SAMHD1 expression of microglia and astrocytes. Cells (1 × 10^6^) were immunostained by FITC-conjugated SAMHD1 monoclonal Ab and analyzed by flow cytometry. Percentage of cells expressing SAMHD1 is indicated. **(F)** Cell culture supernatant from astrocytes and microglia after HIV infection was assessed for viral reverse transcriptase activity over 20 days by colorimetry. **(G)** Total cellular RNA was extracted from the corresponding cells following HIV infection (over a 20-day time course) and SAMHD1 mRNA levels quantitated by RT-PCR. **(H)** SAMHD1 protein expression was detected by immunoblotting with SAMHD1-specific antibodies in corresponding cell lysates at 3, 5, 7, 10, and 15 days following HIV infection. Results shown are mean ± SEM of three independent experiments for A, B, C, F, and G. *P* value ≤0.05 were considered to be significant. SAMHD1, sterile alpha motif and histidine/aspartic acid domain-containing protein 1; GAPDH, glyceraldehyde 3-phosphate dehydrogenase; RT, reverse transcriptase; RLU, relative light units.

To better understand the mechanism behind HIV restriction in astrocytes, we infected astrocytes and microglia with HIV-1_Bal_ (100 ng p24) and estimated the viral replication (by measuring p24 Ag level) in the culture supernatant at 3, 5, 7, 10, 15, and 20 days post infection (dpi). HIV-1 infection in astrocytes peaked at day 5 (450 pg/ml), which then diminished at day 7 and remained low from day 10 onwards (Figure 1A). Whereas, compared to astrocytes, microglia had significantly higher p24 level at day 5 (2,000 *vs* 450 pg/ml) (Figure 1A), which further increased and remained high till 20 dpi. Our observation is in agreement with previous reports, which showed that HIV-1 infection of astrocytes results in an initial productive, non-cytopathogenic infection that diminishes to a poorly productive, persistent infection leading to a latent state [1,2,32]. We also conducted the infection using pseudotyped virus. A lentiviral packaging system was used to generate vesicular stomatitis virus (VSV)g Env-pseudotyped viral particles that contained a HIV-1-integrating provirus (devoid of Env and encoding a luciferase reporter). VSV-G pseudotyped virus can enter CD4 negative cells and thus circumvent the entry restriction in cells lacking CD4 receptors. Compared to microglia, astrocytes have less relative light units (RLU) (Figure 1B) suggesting that even if the entry restriction is bypassed, there are post-entry blocks to HIV infection in astrocytes.

### Astrocytes display higher SAMHD1 expression

Though SAMHD1 has been shown to be responsible for the non-susceptibility of myeloid cells, resting CD4 T cells and macrophages to HIV infection [26,27], its expression has not been evaluated in CNS cells yet. Here, we compared the expression of SAMHD1 in two primary CNS cell types (astrocytes and microglia), which are differentially infected by HIV. We performed real-time PCR analysis to quantify SAMHD1 mRNA expression. By comparing SAMHD1 expression in these two cell types, we expected to see a differential expression of SAMHD1 in non-productively (astrocytes) and productively (microglia) infected cells. As expected, we found that constitutive expression of SAMHD1 mRNA was sevenfold higher in astrocytes compared to microglia (Figure 1C). To further confirm, if the gene expression observed corresponds to protein expression, we performed immunoblotting to detect SAMHD1 protein levels. Similar to gene expression pattern, astrocytes displayed increased SAMHD1 protein expression (Figure 1D). This was further confirmed by intra cellular expression by flow cytometry (Figure 1E).

### HIV replication kinetics correlates with SAMHD1 expression

SAMHD1 hydrolyzes dNTPs and lowers their concentrations to a level that is suboptimal for reverse transcription of viral RNA [25]. We asked whether the SAMHD1 expression plays a role in the restriction of HIV replication in astrocytes and examined whether SAMHD1 expression is modulated by HIV infection. Reverse transcriptase activity measurement was done in culture supernatant at 3, 5, 7, 10, 15, and 20 dpi. The reverse transcriptase assay estimates the quantitative determination of reverse transcriptase (RT) activity. Consistent with p24 level, we found higher reverse transcriptase activity in astrocytes at day 5 post infection, which decreased thereafter (Figure 1F). Reverse transcriptase activity was significantly higher in microglia compared to astrocytes at all-time points (Figure 1F). This suggests that low reverse transcriptase activity contributes towards the restriction of HIV infection observed in astrocytes. Subsequently, we analyzed SAMHD1 mRNA expression in the cells at these same time points following infection and compared with baseline. SAMHD1 mRNA expression in astrocytes was higher at 3 dpi compared to baseline. At day 5, SAMHD1 expression was decreased, which further increased on day 7 and remained high thereafter (Figure 1G). Interestingly, in astrocytes, SAMHD1 gene expression was lowest at day 5, when the p24 level was the highest. SAMHD1 protein expression was also lower on day 5 (Figure 1H). Even though microglia did not show much significant change in the SAMHD1 mRNA expression during the course of infection, the increase in SAMHD1 protein expression was more prominent than gene expression during HIV infection (Figure 1H).

### SAMHD1 silencing relieves the ‘restriction’

**Figure 2:**
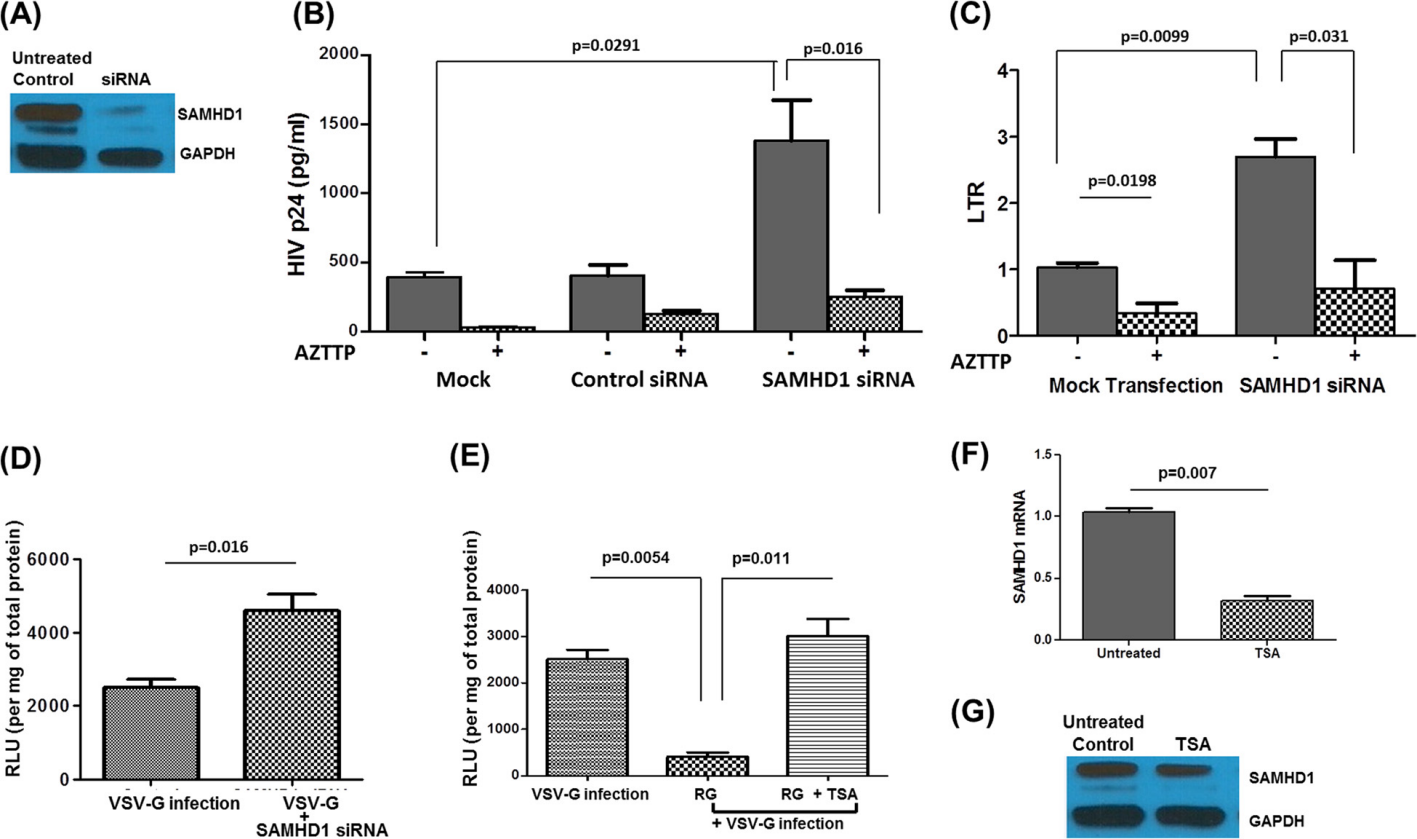
**SAMHD1 silencing promotes HIV replication in astrocytes. (A)** Astrocytes (2 × 10^5^ cells/ml) were transfected with 75 pmols SAMHD1-specific siRNA using Lipofectamine 2000. SAMHD1-knockdown was assessed by immunoblotting with SAMHD1-specific antibodies. One representative blot is shown. **(B)** SAMHD1-siRNA or control siRNA transfected astrocytes were infected by HIV (100 ng p24). Cells were treated with or without 100 nm AZTTP. After 72 h, p24 was measured in supernatants by ELISA. **(C)** Total RNA was extracted from corresponding cells from ‘B’ and amplified by RT-PCR for HIV-1 LTR/RU-5 gene. **(D)** SAMHD1- siRNA transfected astrocytes were infected with the HIV-luciferase pseudovirus (20 ng) for 3 days, harvested and lysed. Luciferase concentration in whole cell lysates was determined. **(E)** Astrocytes were treated with or without raltegravir (10 μM) for 6 h followed by TSA (50 nM) treatment. Cells were infected with the HIV-luciferase pseudovirus (20 ng) for 3 days. Luciferase concentration in whole cell lysates was determined. **(F)** Astrocytes (1 × 10^6^ cells/ml) were treated with trichostatin A (TSA; 50 nM) for 24 h or left untreated. Following the treatment, SAMHD1 mRNA levels were quantified by real-time PCR analysis. The SAMHD1 mRNA level in cells without TSA was set to 1. *P* value ≤0.05 were considered to be significant. **(G)** Cell lysate was prepared from the corresponding cells in **(F)**, and SAMHD1 protein expression was detected in corresponding cell lysates by immunoblotting with SAMHD1-specific antibodies. Equal amounts of total protein (30 μg) from both cells were loaded. GAPDH was used as a loading control. Results shown are representative of mean ± SEM of three independent of experiments (except A and G). *P* value ≤0.05 were considered to be significant. SAMHD1, sterile alpha motif and histidine/aspartic acid domain-containing protein 1; LTR, long terminal repeat; AZTTP, azidothymidine triphosphate; TSA, trichostatin A; VSV-G, vesicular stomatitis virus; RLU, relative light units; GAPDH, glyceraldehyde 3-phosphate dehydrogenase.

To further confirm the role of SAMHD1 in the astrocytic restriction, we evaluated whether SAMHD1 modulation is sufficient to change the viral replication. We transfected the astrocytes (0.5 × 10^6^) with SAMHD1 siRNA (70 nm) to silence SAMHD1 expression, followed by HIV infection. The efficiency of siRNA-mediated silencing was confirmed by strong inhibition of SAMHD1 protein as determined by Western blot analysis (Figure 2A). When we estimated p24 level in the culture supernatant after 72 h post infection, we found significantly higher HIV replication with SAMHD1 silencing (p24 Ag production increased from 390 ± 37.86 to 1,383 ± 169.1 pg/ml) compared to mock transfected cells (Figure 2B). Transfection with negative control siRNA (which consists of a scrambled sequence that will not lead to the specific degradation of the target) did not have any change in infection in astrocytes. This result substantiates that depletion of cellular SAMHD1 in astrocytes is sufficient to alleviate the restriction to HIV infection.

In order see if the SAMHD1-mediated repression of HIV-1 replication is due to its ability to repress reverse transcription, we evaluated whether the process of reverse transcription occurred efficiently following SAMHD1 silencing. Infected astrocytes were analyzed by qPCR for early reverse transcription products at 72 h post infection using a set of primers specific for long terminal repeat (LTR) R/U5 (short first transcript). Sequences of PCR primers have been previously described [33]. We saw that SAMHD1 inhibition increased LTR R/U5 approximately 2.5-fold (Figure 2C). Negative control siRNA transfection did not show any difference in the LTR R/U5 expression. These results indicate that SAMHD1 has an effect at the reverse transcription phase in astrocytes. Interestingly, SAMHD1 silencing did not show any significant change in the p24 in microglia (not shown).

We treated the astrocytes with a reverse-transcriptase inhibitor, azidothymidine triphosphate (AZTTP, 100 nm) 6 h before siRNA transfection. Treatment with AZTTP significantly inhibited the p24 production in infected astrocytes (390 ± 37.86 to 30.67 ± 3.48, 93% decrease; Figure 2B). SAMHD1 siRNA-mediated increase in the p24 level was also inhibited by AZTTP (1,383 ± 169.1 to 250.3 ± 28.87 pg/ml, 82% decrease). Similar to the observation in p24 level, AZTTP treatment suppressed SAMHD1 siRNA-mediated increase in LTR R/U5 expression (Figure 2C). Further, we infected the astrocytes with VSV-G pseudotype virus following SAMHD1 siRNA transfection. We found approximately two fold increase in luciferase activity after SAMHD1 siRNA transfection (Figure 2D). This further confirms that SAMHD1 has a role in suppression of HIV-1 replication in astrocytes, even though the virus gets rid of the entry restriction. This is in accordance with the previous reports in resting CD4 T cells and myeloid cells explaining that SAMHD1 is responsible for the efficient reverse transcription [27,34,35]. These results confirm the role of SAMHD1 as a restriction factor that facilitates in HIV-1 restriction in astrocytes and silencing SAMHD1 relieves the ‘restriction.’ Additional investigations are needed to confirm that the restriction is at the level of reverse transcription. However, the possibility of additional SAMHD1-dependent/independent mechanisms cannot be excluded.

### Reactivation decreases SAMHD1 expression

Neutralizing the miRNAs or histone deacetylase (HDAC) inhibitors treatment in latently infected cells has shown to revert the latency and reactivate the HIV infection [36-38]. We wanted to see if the reactivation of virus is accompanied by change in SAMHD1 expression. An *in vitro* model mimicking latent infection in astrocyte was prepared as described by Gray *et al*. to make sure the cells were in latent stage before trichostatin A (TSA) treatment [39]. Astrocytes were infected with the pseudotyped virus in the presence or absence of the integrase inhibitor raltegravir for 2 days. Cells were then treated with TSA and reactivation from latency was assessed by measuring luciferase activity. As shown in Figure 2E, TSA treatment induced reactivation from raltegravir-treated cells. This was in agreement with the abovementioned reports, which showed an increase in HIV replication with TSA treatment. We further evaluated the SAMHD1 expression in TSA treated cells. We found that TSA treatment was accompanied by a decrease in the SAMHD1 expression both in gene and protein level (Figure 2F, G).

### miR-181a and −155 targets SAMHD1 in astrocytes

Neutralizing the miRNAs in latently infected cells has shown to revert the latency [38]. Yet, the information available about role of miRNAs in CNS HIV replication is limited. Moreover, mechanism of SAMHD1 regulation is also not known. Since both miRNAs and SAMHD1 plays a role in HIV replication, we asked whether SAMHD1-mediated HIV-1 restriction in astrocytes is regulated by miRNA-mediated translational inhibition. We screened web-based tools TargetScan (http://www.targetscan.org/) and http://www.mirdb.org to identify potential miRNAs that can target SAMHD1 (by searching for the presence of conserved 8mer and 7mer sites that match the seed region of each miRNA). We were able to identity few miRNAs (miR-124, −150, −155, −181a-d, −490, and −496) that have binding sites on SAMHD1. We concentrated our further experiments on miR-155 and -181a, because of their importance in the brain and latency [40-43].

**Figure 3:**
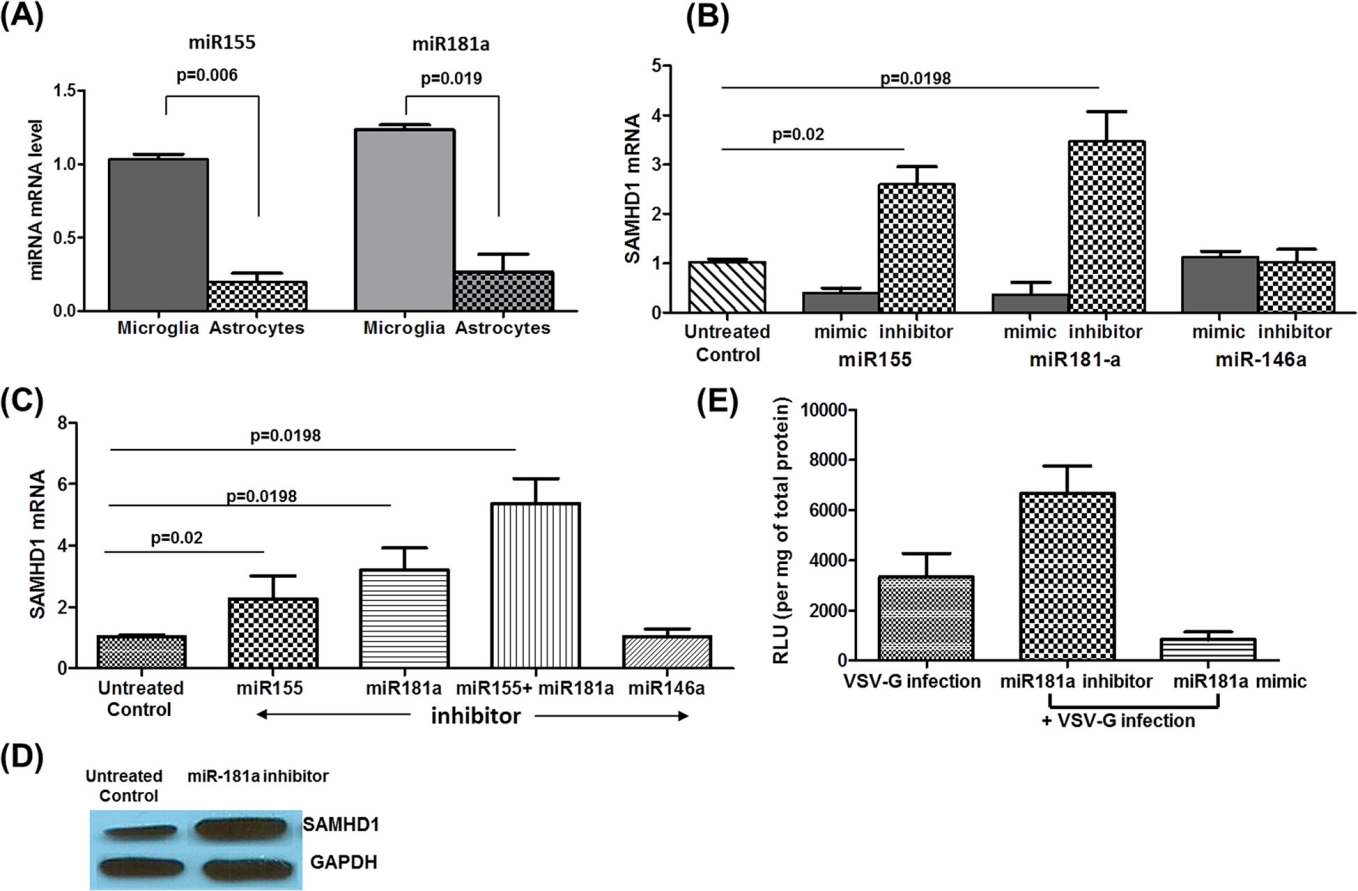
**miR-155 and -181a target SAMHD1. (A)** Quantification of miRNA levels. Total cellular microRNA was extracted from microglia and astrocytes and amplified for miRNA expression by qRT-PCR using specific primer for miR-155 and miR-181a. miRNA levels were normalized to U6 snRNA control and the cycle threshold (Ct) values were calculated. **(B)** Astrocytes (2 × 10^5^ cells/ml) were transiently transfected with mimic or inhibitor for miR-155, miR-181a, and miR-146a, at a final concentration of 50 nmol. After 72 h, total cellular RNA was extracted and amplified by specific primer for SAMHD1 gene expression by RT-PCR. All data was normalized to GAPDH. Data shown represents fold change in mRNA levels. **(C)** Astrocytes (2 × 10^5^ cells/ml) were transiently transfected with inhibitor for miR-155, miR-181a, or miR-146a individually or a combined transfection with both miR-155 and miR-181a inhibitors. Following transfection, SAMHD1 mRNA levels were quantified by RT-PCR. **(D)** Lysates were prepared from miR-181a inhibitor transfected cells and SAMHD1 protein expression was detected in corresponding cell lysates by immunoblotting with SAMHD1-specific antibodies. Equal amounts of total protein (30 μg) of the lysates from both cells were loaded. GAPDH was used as a loading control. **(E)** Astrocytes (2 × 10^5^ cells/ml) were transiently transfected miR-181a inhibitor/mimic at a final concentration of 50 nmol. After 12 h, cells were infected with the HIV-luciferase pseudovirus (20 ng). At day 3, cells were harvested, washed with PBS, and lysed. Luciferase concentration in whole cell lysates was determined. Results shown are representative of mean ± SEM of three independent experiments (except for **(D)**). *P* value ≤0.05 were considered to be significant. SAMHD1, sterile alpha motif and histidine/aspartic acid domain-containing protein 1; VSV-G, vesicular stomatitis virus; GAPDH, glyceraldehyde 3-phosphate dehydrogenase.

To verify the relatively abundant expression of these two miRNAs, we measured constitutive expression of miR-181a/-155 in astrocytes and microglia by TaqMan assay (Life Technologies, Carlsbad, CA, USA). We saw significantly lower constitutive expression of miR-181a and miR-155 in astrocytes (in which SAMHD1 expression is higher) compared to microglia (Figure 3A). The level of both miRNAs was comparable in astrocytes. Our next aim was to assess the inhibitory effects of miR-181a and miR-155 on SAMHD1 expression and to verify that the SAMHD1 is a direct target of these identified miRNA sequences. We transfected astrocytes with specific miR-181a or miR-155 inhibitor/mimic (Ambion) for 72 h using Lipofectamine (Invitrogen, Carlsbad, CA, USA) and analyzed the SAMHD1 expression. miR-146a, a non-targeting miRNA, was used as control. qPCR analysis revealed an increase in SAMHD1 mRNA expression with miR-155 (2.6-fold) and miR-181a inhibition (3.4-fold), compared to non-transfected cells, miR-181a being more effective (Figure 3B). Although individual inhibitors increased SAMHD1 only modestly, the combination of these two miRNA inhibitors resulted in a substantial increase in SAMHD1 expression (sixfold higher than the control) in astrocytes (Figure 3C). On the other hand, over expression of miR-181a and miR-155 by respective mimics suppressed the SAMHD1 mRNA expression in astrocytes (Figure 3B). Non-targeting miRNA (miR-146a) mimic/inhibitor did not have any effect on SAMHD1 expression. Given the significant induction of SAMHD1 mRNA in astrocytes with miRNA inhibition, immunoblotting was performed to examine whether the increase in SAMHD1 mRNA resulted in the expression of SAMHD1 protein. SAMHD1 protein was also induced with miR-181a inhibition compared to untreated control (Figure 3D). miR-181a inhibitor increased the luciferase activity in VSV-G pseudotype virus infection and miR-181a mimic transfection decreased the luciferase activity (Figure 3E). These results illustrate that transfection with miR-181a/miR-155 inhibitor and mimic modulated the SAMHD1 expression confirming that SAMHD1 is regulated by miR-181a and miR-155.

### miR-181a/miR-155 inhibition increases HIV replication

**Figure 4:**
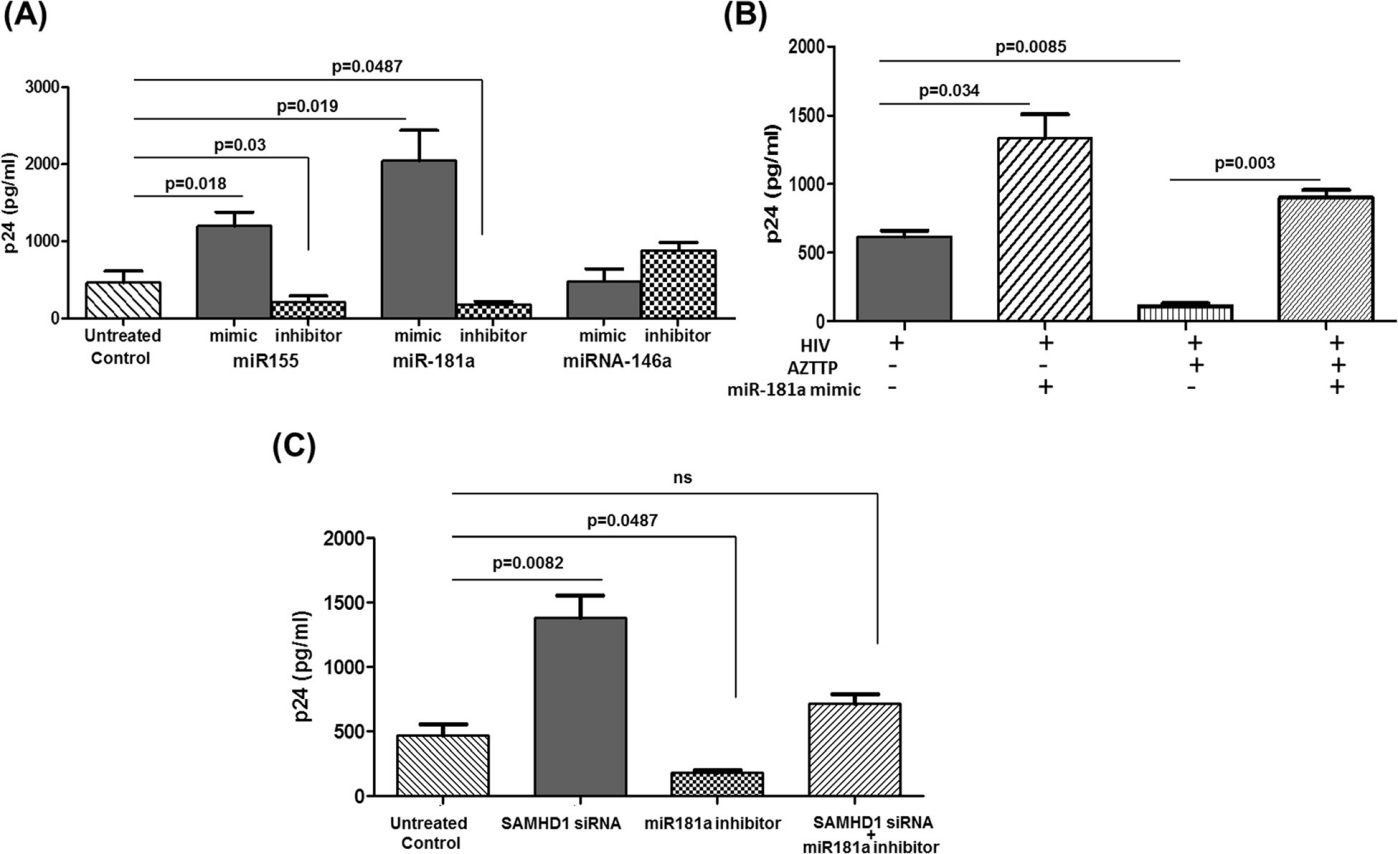
**miR-155/-181a inhibition increases HIV replication. (A)** Astrocytes (2 × 10^5^ cells/ml) were transiently transfected with mimic or inhibitor for miR-155, miR-181a, and miR-146a, at a final concentration of 50 nmol, followed by HIV infection. After 72 h, supernatants were collected and assayed for p24 by ELISA. **(B)** Astrocytes (2 × 10^5^ cells/ml) were transiently transfected with miR-181a mimic, followed by infection with HIV-1_BAL_ (100 ng p24) with or without 100 nm AZTTP. p24 level was measured in the supernatant from astrocytes cultures. **(C)** Astrocytes (2 × 10^5^ cells/ml) were transiently transfected with SAMHD1 siRNA (75 pmols) and miR-181a inhibitor (50 nmols) individually or a combined transfection with both. After 72 h, supernatants were collected and assayed for p24 by ELISA. Results shown are representative of mean ± SEM of three independent experiments. *P* value ≤0.05 were considered to be significant. SAMHD1, sterile alpha motif and histidine/aspartic acid domain-containing protein 1; AZTTP, azidothymidine triphosphate.

Further, we wanted to verify that the identified putative miRNAs have roles in the suppression of HIV-1 protein expression. We transfected astrocytes with miRNA mimic/inhibitor as described above and cultured with HIV for 72 h. p24 production was measured in the culture supernatants. We found that miR-181a and miR-155 overexpression displayed an increase in p24, whereas their inhibition resulted in p24 reduction (Figure 4A). Over expression of non-targeting miRNA (miR-146a) did not have any significant change on p24 level. Interestingly, miR146a inhibition resulted in a slight increase. Reduction in p24 by reverse-transcriptase inhibitor (AZTTP) treatment was abrogated by miR-181a overexpression (Figure 4B). Our data indicated that overexpression of these two miRNAs by their corresponding mimics increases HIV-1 production from astrocytes, suggesting that miRNA overexpression alleviates the restriction. Further investigations are necessary to confirm whether these miRNA can reactivate productive infection in the astrocytes.

Further, we wished to determine whether miRNA(s) have a direct role in the regulation of HIV-1 gene expression or whether the miRNA-mediated regulation of HIV-1 replication is through its ability to modulate SAMHD1. We co-transfected SAMHD1 siRNA and miR-181a inhibitor in astrocytes, followed by HIV infection. As shown in previous experiment, SAMHD1 silencing increased the p24 level, whereas miR-181a inhibition decreased p24 level (Figure 4C). Interestingly, co-transfection did not have significant change in p24 level compared to the untreated control. This data confirms that miR-181a/miR-155-mediated HIV regulation is by inhibiting SAMHD1.

## Discussion

Astrocytes, which constitute 40% to 70% of total cells in the CNS [1], perform key regulatory functions critical to brain function. The different CNS cell types are differentially infected with HIV; microglia being highly susceptible, astrocytes moderately restrictive, and neurons highly restrictive. Despite the lack of CD4 receptors, astrocytes become infected via CD4-independent mechanism [6,44]. In line with the earlier studies [1,2], we also found low level of HIV replication in astrocytes compared to microglia. After the initial productive phase, infection subsides to a persistent stage in astrocytes, which goes in hand with reports from other investigators [1,2,32]. Infected astrocytes produce very low levels of virus even in the acute phase in contrast to infection of T-lymphocytes [45,46]. Our results with pseudotyped virus confirm that regardless of the entry routes, there are post-entry blocks to infection in astrocytes. Though the introduction of potent HAART has significantly controlled the viral replication in HIV patients, the current antiviral strategies target only actively replicating virus. Having said that, persistently infected brain astrocytes would not be susceptible to antiviral drugs, allowing the virus to persist in these reservoirs. Hence, the complete eradication of the virus from the host remains an impossible task. Several post-transcriptional and post-translational mechanisms [19,20], a number of host restriction factors [21,22], and certain cellular miRNAs [24] have been reported to hinder retroviral replication in astrocytes contributing to HIV latency.

SAMHD1, a recently identified nuclear protein restriction factor, is highly expressed in HIV-1 non-permissive cells, whereas it is absent from HIV-1-sensitive T-cell lines such as Jurkat, SupT1, human peripheral blood acute lymphoid leukemia, and U937 [27]. Since SAMHD1 has not been studied in CNS cells, our aim in this study was to explore the role of SAMHD1 in CNS cells. And we found that astrocytes and microglia express differential endogenous levels of SAMHD1, suggesting that possibly the higher level of SAMHD1 contribute towards HIV restriction in astrocytes. Ours is the first study presenting the role of SAMHD1 in astrocytes.

We found higher reverse transcriptase activity in microglia compared to astrocytes, suggesting low reverse transcriptase activity may be responsible for the restriction of HIV infection observed in astrocytes. Our results also substantiate that decrease in viral replication in astrocytes was in correlation with the increased expression of SAMHD1 and depletion of cellular SAMHD1 in astrocytes is sufficient to alleviate the restriction to HIV infection. This suggests that replication kinetics of the virus follows an inverse relation with SAMHD1 and confirms that SAMHD1 is significantly regulated over the course of infection in astrocytes. Interestingly, SAMHD1 silencing did not show any significant change in the infection in microglia. Our results support the role of SAMHD1 as a restriction factor that facilitates in HIV-1 restriction in astrocytes. Since the role of SAMHD1 is to deplete dNTPs required for RT, we feel that SAMHD1-mediated repression of HIV-1 replication in astrocyte is also due to its ability to repress RT. This goes in hand with the previous reports of SAMHD1 responsible for suboptimal reverse transcription of viral RNA in T cells and DCs [25]. However, SAMHD1 may not be the only restriction factor playing a role in astrocytes. The possibility of additional SAMHD1-dependent/independent mechanisms cannot be excluded.

Complete eradication of HIV is possible only if HAART regimens totally stop all new infections of susceptible cells, along with flushing out existing reservoirs and blocking formation of additional long-lived viral reservoirs or reactivating reservoirs in chronically infected patients on HAART [47,48]. To reactivate viral replication, it is necessary to understand the regulatory mechanisms involved in establishing persistent reservoirs. We found that reactivation of viral replication by HDAC inhibitor, TSA, is accompanied by decrease in SAMHD1 expression. This was in contrast to a recent report showing increase in SAMHD1 expression in CD4-T cells with TSA exposure [49]. De Silva *et al*. indicated in their study that HDAC inhibition did not significantly change the SAMHD1 protein expression [28]. Though we are not sure the reason for this difference, this suggests that SAMHD1 may be subjected to post-transcriptional regulation. Even though, the restricted HIV-1 replication in myeloid cells, resting T-cells and macrophages has been linked to SAMHD1 expression, little is known about its regulatory mechanism. So, in our next set of experiments, we focused our attention on the molecular mechanism of SAMHD1 regulation.

The miRNAs induce mRNA cleavage or translation repression by targeting complementary or partly complementary sequence in the 3′-untranscribed region (UTR) of target mRNAs [50]. Certain cellular miRNAs have a physiological role in controlling HIV-1 replication and in the maintenance of HIV-1 latency in the peripheral cells [29-31]. We found six miRNAs (miR-124, −150, −155, −181a-d, −490, and −496) to have binding site on SAMHD1, suggesting that SAMHD1 may be regulated by these miRNAs. miR-181 is reported to be very strongly expressed in the brain [41-43], downregulation of which contributes to accelerated HIV-associated dementia [51], and miR-155 is reportedly absent from latently infected cells [30]. Hence, we concentrated our further experiments on these two miRNAs. In our efforts to verify the role of these miRNAs in the regulation of SAMHD1 and HIV, we saw that modulation of these miRNAs had an impact on the expression of SAMHD1 and also on HIV-1 replication. Combination of these two miRNA inhibitors resulted in a substantial increase in SAMHD1 expression. Interestingly, miR146a (non-targeting miRNA) inhibition resulted in a slight increase, which was unexpected. This may be because, miR146a controls HIV replication through some other means (other than SAMHD1). The restriction in the astrocytes was alleviated miRNA overexpression, which was mediated by the change in SAMHD1 expression. Viral miR-K12-11, an orthologue of cellular miR-155, has been reported to target SAMHD1 [52], which goes in hand with our finding. To the best of our knowledge, ours is the first study showing miR181a regulation of SAMHD1 in astrocytes.

In summary, the importance of our study is that it provides a proof of concept that SAMHD1 facilitates HIV-1 restriction in astrocytes and miR-181a/miR-155 regulates SAMHD1 expression. Overexpression of these cellular miRNAs or suppression of SAMHD1 removed the inhibition of HIV-1 replication resulting in increased viral production from the astrocytes. As a result, this may allow persistently infected astrocytes to be exposed to immune surveillance or for the action of HAART. At this time, this concept is of theoretical nature, as we did not have any *in vivo* data. Future *in vivo* experiments are needed to evaluate if optimal combinations of miRNA or SAMHD1 siRNA delivered to astrocytes along with HAART could reactivate the HIV replication in persistently latent cells.

## Material and methods

### Cells and reagents

The primary astrocytes (HA #1800) and microglia (HM #1900) types are isolated from normal human brain and are commercially available (Sciencell Research Laboratories, Carlsbad, CA, USA). Cells were grown in the specified media as recommended by the manufacturer. Depending upon the experiments, cells were cultured at a concentration of 5 × 10^5^ cells/ml in six-well plates (for transfection experiment) or 1 × 10^6^ cells/ml and allow them to reach at least 70% confluency before any further treatment.

### HIV infection

Microglia and astrocytes (1 × 10^6^ cells/ml) were infected with HIV-1_Bal_, (NIH AIDS Research and Reference Reagent Program; catalog no. 510) at a concentration of HIV-1 p24 100 ng/10^6^ cells for 24 h. Cells were washed to remove unbound virus and replenished with fresh media.

### SAMHD1 mRNA quantification

For SAMHD1 mRNA quantification, total cellular RNA was extracted using the RNeasy mini kit (Qiagen, Limburg, the Netherlands) according to the manufacturer’s guidelines. One hundred nanograms of the total RNA from each cell type was used as a template for first-strand cDNA synthesis using high-capacity reverse transcriptase kit (Applied Biosystems, Waltham, MA, USA, #4368814). SAMHD1 mRNA was quantitated by SYBER Green-based quantitative real-time PCR (Stratagene-3000; Stratagene, La Jolla, CA, USA) analysis was performed using the specific primers. Quantification of glyceraldehyde 3-phosphate dehydrogenase (GAPDH) mRNA was carried out for normalization. Relative expression of mRNA species was calculated using the comparative *C*_T_ method.

### Immunoblotting of SAMHD1

To assess SAMHD1 protein levels, cells were harvested after respective treatments and/or transfection (supernatant was collected for the detection of p24 and reverse transcriptase activity assay). Cells were washed twice with PBS and homogenized in cell lysis buffer (MPER mammalian protein extraction reagent, Thermo Scientific, Waltham, MA, USA) supplemented with protease inhibitor mixture (Thermo Scientific, Waltham, MA, USA) by incubating on ice for 15 min. The homogenates were centrifuged at 10,000 rpm for 10 min and the supernatant was used for further analysis. Protein quantification was carried out by using Bio-Rad protein assay kit (Bio-Rad Laboratories Inc., Hercules, CA, USA). Thirty micrograms of whole cell lysate was electrophoretically separated on an SDS-polyacrylamide gel. Separated proteins were transferred to nitrocellulose paper for 90 min at 100 V, using a wet electroblotting system (Bio-Rad Laboratories Inc., Hercules, CA, USA). Blots were blocked for 1 h in 5% non-fat dry milk in Tris-buffered saline-Tween (TBST) (20 mM Tris, 150 mM NaCl, 0.1% Tween 20, pH 7.5), followed by incubation overnight with the primary antibody (1:500 SAMHD1 polyclonal antibody; # sc-86212, Santa Cruz Biotechnology, Inc., Dallas, Texas, USA) in 1% milk solution. After several washes in TBST, the membranes were incubated in TBST/2% non-fat dry milk, containing the donkey anti-goat coupled to horse radish peroxidase (1:50,000 dilution; #sc-2020, Santa Cruz Biotechnology, Inc., Santa Cruz, CA, USA) for 1 h at RT. After washes in TBST, immunoreactivity was visualized using Western blotting detection reagent (SuperSignal West Pico enhancer, Thermo Scientific, Waltham, MA, USA). Expression of GAPDH (monoclonal mouse, Sigma-Aldrich, St. Louis, MO, USA, 1:50,000) was used as loading control.

### SAMHD1 siRNA transfection

SAMHD1-siRNA and scrambled siRNA were procured from Santa Cruz Biotechnology. Astrocytes (2 x 10^5^) were seeded into antibiotic-free Dulbecco's Modified Eagle’s medium (DMEM) supplemented with 10% FBS 24 h before transfection. On day of transfection, 75 pmols siRNA duplex and 6 μl of siRNA transfection reagent (sc-29528) are diluted into 100 μl serum and antibiotic-free siRNA transfection medium (sc-36868) in separate tubes. Both the solutions are mixed gently and incubated for 30 min at room temperature. This mixture is diluted with 0.8 ml siRNA transfection medium, overlayed onto the cells and incubated at 37°C in a CO_2_ incubator. After 5 h, cells are supplemented with 1 ml of normal growth medium containing two times the normal serum and antibiotics concentration without removing the transfection mixture. Cells were left untreated on infected with HIV and incubated for another 72 h. A second round of transfection was performed 24 h after the first transfection, whenever it was needed. Cells were harvested for RNA or protein isolation, and supernatant collected for p24 and RTase measurements.

### miRNA expression assay

Total RNA was isolated using the mir-Vana miRNA isolation (#:1560; Ambion), in accordance with the manufacturer’s protocol. RNA concentrations were determined and 2 ng of each RNA sample were used for cDNA synthesis using TaqMan MicroRNA reverse transcription kit. Quantitative real-time RT-PCR (qRT-PCR) was performed using specific primers for miR-155 and miR-181a (Applied Biosystems, Waltham, MA, USA). All reactions were analyzed using StepOne Real-Time PCR System (Applied Biosystems, Waltham, MA, USA). miRNA levels were normalized to U6 snRNA control and the cycle threshold (Ct) values were calculated.

### miRNA transfection

Cells in 12-well plates were transfected either with anti-miR inhibitor or miRNA mimic (Applied Biosystems, Waltham, MA, USA) in inhibition or in overexpression experiments respectively, using Lipofectamine according to the manufacturer’s instructions. Briefly, 5 ul Lipofectamine was added to 200 ml of serum free medium (DMEM + F10) per condition for 5 min followed by incubation with anti-miR or miR-mimic at a final concentration of 50 nM. The volume was adjusted to 1 ml with medium containing 10% FCS and added to the cells. Following transfection, cells were treated with HIV or left uninfected for 72 h after which the cells were harvested for RNA or protein isolation, and supernatant collected for p24 and RTase measurements.

### HIV LTR

Following HIV infection/transfection, cells were collected, RNA isolated (RNAeasy mini kit; Qiagen, Limburg, the Netherlands) and qRT-PCR run to amplify a 180 bp fragment in the LTR-R/U5 region that represents early stages of reverse transcription of HIV-1 using the following primers: 5′-primer, 5′-TCTCTCTGGTTAGACCAGATCTG; 3′-primer, and 5′-ACTGCTAGAGATTTTCCACACTG.

### Reverse transcriptase activity

Reverse transcriptase activity of samples was assayed using a colorimetric reverse transcriptase assay (Roche Applied Science, Basel, Switzerland). Supernatants were collected at the end of infection, and the viruses were pelleted by ultra-centrifugation at 100,000 *g* for 120 min at 4°C. Virus pellets were resuspended and lysed by adding 40 μl lysis buffer, followed by addition of 20 μl of mixture containing template/primer hybrid and nucleotide, and incubated for 15 h at 37°C. Samples were then processed as per kit protocol for ELISA and absorbance read at 405 nm. Reactions were performed in triplicate, and experiments were performed thrice and the results averaged.

### p24

p24 Ag concentration in the culture supernatants were assayed in an ELISA method using ZeptoMetrix RETRO-TEK ELISA kit (ZeptoMetrix Corporation, Buffalo, NY, USA) as per manufacturer’s recommendations.

### TSA treatment

For the TSA (Cell Signaling, Danvers, MA, USA) treatment, after reaching confluency, the cells were with 50 nM TSA for 24 h and washed and proceeded with cell isolation for RT-PCR and Western blot.

### Flow cytometry

Cells (1 × 10^6^) were permeabilized with perm wash buffer and stained with fluorescein isothiocyanate (FITC)-conjugated SAMHD1 monoclonal ab (Abcam plc, Cambridge, MA, USA; #ab128107) at 4°C for 20 min, and washed. Samples were acquired using FACSCalibur (BD Biosciences, San Jose, CA, USA) with collection of 100,000 cells and analyzed using FlowJo software. SAMHD1 ab was conjugated to FITC by EasyLink FITC Conjugation Kit (Abcam plc, Cambridge, MA, USA; #ab102885) as per the kit protocol.

### Plasmids, transfections, and luciferase assay

The following plasmids were obtained through the NIH AIDS Reagent Program, Division of AIDS, NIAID, NIH: pHIV-CAT (cat#2619) from Dr. Gary Nabel and Dr. Neil Perkins; pSVTat (cat#294) from Dr. Alan Frankel; pNL4-3-Luc.R^−^E^−^(cat#3418) Dr. Nathaniel Landau, Aaron Diamond AIDS Research Center, The Rockefeller University; and pHEF-VSV-G from Dr. Lung-Ji Chang. Amphoteric HIV pseudoviruses containing the firefly luciferase gene were produced by transfecting 293 T cells with pHEF-VSV-G (10 μg) and pNL4-3. Luc.R-E- (10 μg) in 90-mm culture dishes. Briefly, plasmid DNA was diluted in Opti-MEM serum free media (Invitrogen, Carlsbad, CA, USA). Lipofectamine LTX and PLUS reagent (Invitrogen, Carlsbad, CA, USA) diluted in equal volume and added to diluted DNA at a 1:1 ratio. Mixture was incubated for 5 min to allow formation of DNA-liposome complexes. Culture supernatants containing the pseudoviruses were collected 72 h post-transfection and clarified by centrifugation at 400 *g*. Pseudoviruses were competent for single round of replication. Astrocytes infected with the HIV-luciferase pseudovirus (20 ng) were harvested 3 dpi, washed with PBS and lysed. Lysates were pre-cleared by centrifugation at 12,000 *g* for 2 min at 4°C. Luciferase concentration in whole cell lysates from cells was determined using a Luciferase assay system (Promega, Madison, WI, USA), according to the manufacturer’s instructions (Technical Bulletin #TB281). One hundred microliters of luciferase assay reagent were dispensed in a 96-well microplate (Costar, Corning, NY), 20 μl of cell lysate added to each well, mixed by pipetting, and read on a luminometer (Synergy HT, BioHIT, Sartorius AG, Goettingen, Germany).

### Statistical analysis

Results are presented as mean ± SE of three independent experiments. Differences between treated and untreated cultures were compared by Student’s *t*-test. Comparisons of more than two groups were made using analysis of variance (ANOVA) (Kruskal-Wallis). Data were analyzed using Graphpad Prism version 5.0 software and statistical significance considered when *P* value ≤0.05.
